# Pulmonary Mucoepidermoid Carcinoma With Multiple Paraneoplastic Syndromes

**DOI:** 10.7759/cureus.44193

**Published:** 2023-08-27

**Authors:** Sok Boon Tay, Issam Al Jajeh, Lianne Ai Ling Lee, Lynette Lee

**Affiliations:** 1 Department of Respiratory Medicine, Sengkang General Hospital, Singapore, SGP; 2 Department of Pathology, Sengkang General Hospital, Singapore, SGP; 3 Department of Endocrinology, Sengkang General Hospital, Singapore, SGP

**Keywords:** paraneoplastic leukemoid reaction, ectopic acth syndrome, ectopic cushing’s syndrome, humoral hypercalcemia of malignancy, paraneoplastic syndrome, pulmonary mucoepidermoid carcinoma

## Abstract

Pulmonary mucoepidermoid carcinoma (PMEC) is rare and challenging to diagnose. Its association with paraneoplastic syndromes is poorly described. It is also uncommon for a patient with lung cancer to present with multiple paraneoplastic syndromes. We report a case of a patient with metastatic high-grade PMEC associated with three paraneoplastic syndromes, namely, humoral hypercalcemia of malignancy, ectopic ACTH syndrome, and paraneoplastic leukemoid reaction.

## Introduction

Paraneoplastic syndromes occur in many malignancies, but it is most commonly associated with lung cancers [1]. It has been reported to be found in 11% of all lung cancers [2]. Pulmonary mucoepidermoid carcinoma (PMEC) is a rare salivary-gland-type tumor of the lung, comprising <1% of all lung cancers [3]. It is derived from the minor salivary cells in the tracheobronchial tree and subclassified into low-grade and high-grade tumors.

While certain paraneoplastic syndromes are well described in association with small-cell lung cancer (SCLC) and non-small-cell lung cancer (NSCLC) [4], syndromes associated with PMEC have not been not well established. In addition, multiple paraneoplastic syndromes occurring in the same patient are uncommon, and it may portend a poorer prognosis. We report an interesting case of PMEC associated with three concomitant paraneoplastic syndromes.

## Case presentation

A 74-year-old female presented with a three-month history of central, non-exertional chest pain and unintentional weight loss. There were no infective symptoms. She is a nonsmoker and has no known past medical history apart from mild COVID-19 three months prior, which she recovered uneventfully at home.

**Figure 1:**
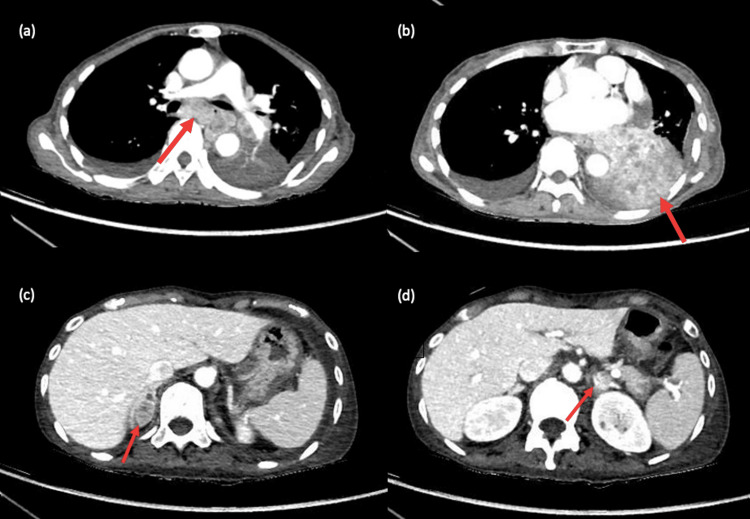
CT thorax, abdomen, and pelvis. (a) Subcarinal lymphadenopathy, (b) necrotic left lower lobe lung mass, (c) right adrenal metastatic nodule, and (d) left adrenal metastatic nodule. CT, computed tomography

**Figure 2:**
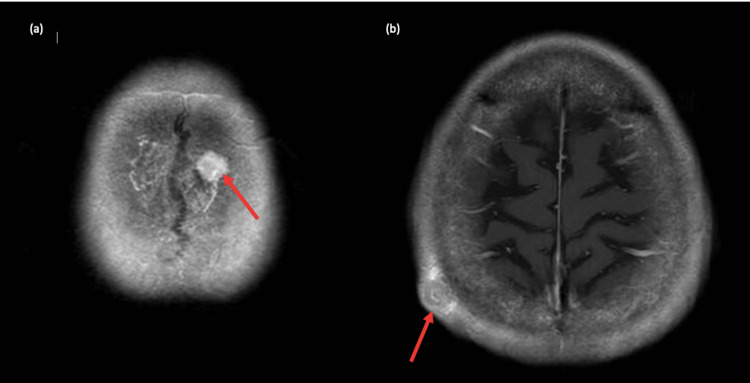
MRI brain: (a) intradiploic metastatic lesion; (b) right parietal metastatic lesion. MRI, magnetic resonance imaging

The chest radiograph showed increased opacification in the left retrocardiac region. She underwent a CT scan of the thorax, abdomen, and pelvis, which reported a 9.1 cm × 6.4 cm left lower lobe necrotic lung mass (Figure 1b); confluent enlarged subcarinal (Figure 1a); and mediastinal and hilar lymph nodes, bilateral pleural effusions, and bilateral necrotic adrenal nodules (Figures 1c-1d) with no destructive bony lesions. The brain MRI exhibited diffuse pachymeningeal and intradiploic enhancement (Figure 2a) and a right parietal soft tissue enhancement (Figure 2b) suspicious for metastasis.

**Figure 3:**
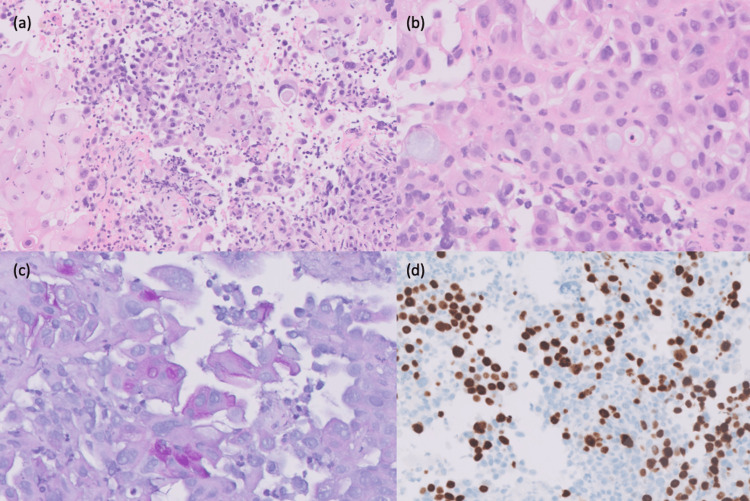
Histology of station 7 lymph node TBNA. (a) Admixture of intermediate, epidermoid, and mucinous cells ×10 scan factor ×40 (hematoxylin-eosin). (b) Scattered cells with basophilic mucin vacuoles ×40 (hematoxylin-eosin). (c) Scattered mucinous cells ×10 scan factor ×40 (periodic acid-Schiff with diastase). (d) Patchy nuclear staining ×10 scan factor ×40 (p40 immunohistochemistry). TBNA, transbronchial needle aspiration

An endobronchial ultrasound (EBUS) and transbronchial needle aspiration (TBNA) of the station 7 lymph node were done. Histological examination showed a poorly differentiated NSCLC, which was mucoepidermoid-like. It comprised a solid growth pattern and typical admixture of intermediate, epidermoid, and mucinous cells (Figures 3a-3b). The latter exhibited carminophilic and PAS-D reactive properties (Figure 3c). Tumor cells tested positive for p40 and CK5/6 while being negative for TTF1, synaptophysin, and chromogranin (Figure 3d). Fluorescence in situ hybridization (FISH) analysis for mastermind-like transcriptional coactivator 2 (MAML2) break apart, as well as epidermal growth factor receptor (EGFR) and anaplastic lymphoma kinase (ALK) tests, yielded negative results.

The differential diagnosis included adenosquamous carcinoma and MAML2-negative, high-grade mucoepidermoid carcinoma. The preference was given to the latter option due to its fitting morphology and immunophenotype, as well as the lack of dual and distinct gland-forming and squamous components characteristic of adenosquamous carcinoma. The sensitivity of the MAML2 mutation test in pulmonary mucoepidermoid carcinoma is low in high-grade mucoepidermoid carcinoma. In a review of 62 patients with PMEC, MAML2 rearrangement was positive in only 18.8% of high-grade tumors [5].

**Figure 4:**
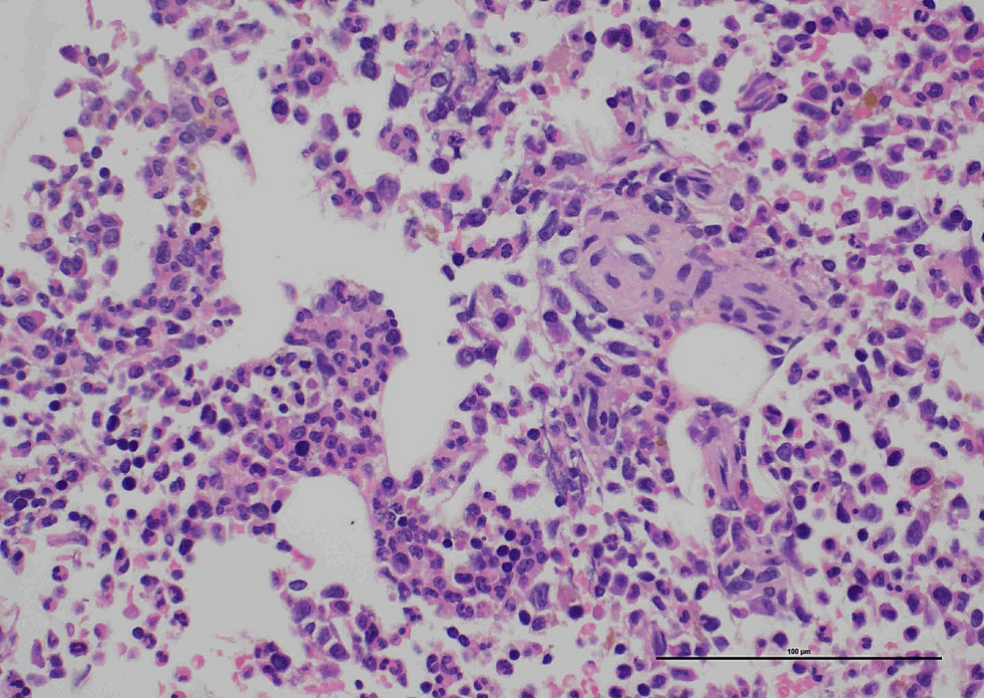
Bone marrow trephine showing increased numbers of mature granulocytes without features of dysplasia.

The initial full blood count (FBC) showed a significantly raised white cell count of 78 × 10^9^ cells/L, which subsequently peaked at 131 × 10^9^ cells/L. No blasts were present on the peripheral blood smear. Bone marrow aspirate and trephine showed increased mature granulocytes without dysplasia, negative aberrant mutations, and no abnormal clonal cells; hence, the diagnosis of a paraneoplastic leukemoid reaction (PLR) was made (Figure 4). As the risk of hyperviscosity syndrome by the mature leukocytes was low, no specific therapy was required.

The patient presented with hypercalcemia (highest serum calcium 3.71 mmol/L) with hypophosphatemia. The parathyroid hormone (PTH) level was below the detectable range. A bone scan did not show any evidence of osteoblastic metastasis. The patient received intravenous (IV) hydration and subcutaneous calcitonin and IV Zoledronate 4 mg once. Calcium levels normalized four days after administration of Zoledronate. The 1,25OH Vitamin D levels came back as normal, but there was an elevation in PTH-related peptide at 4.9 pmol/L, confirming the presence of humoral hypercalcemia of malignancy (HHM).

**Figure 5:**
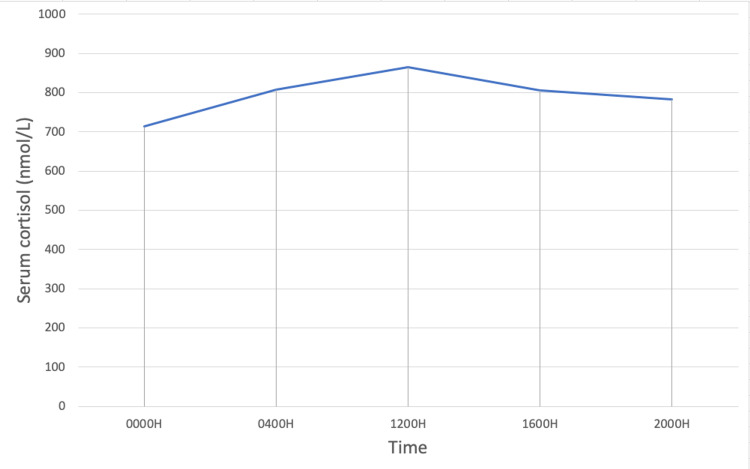
Cortisol day curve demonstrating loss of diurnal variation.

**Figure 6:**
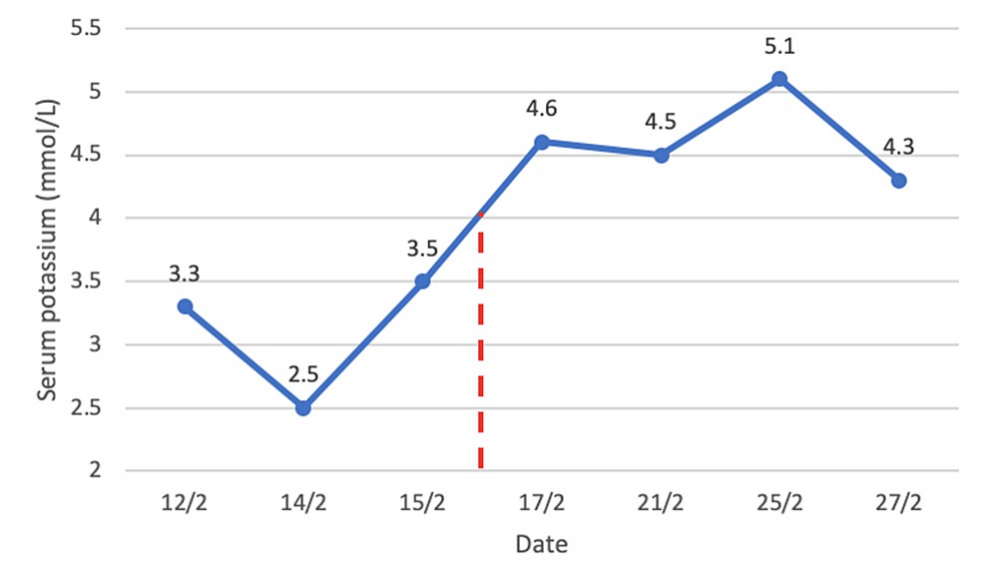
Serum potassium levels, with the red line indicating the commencement of Ketoconazole treatment.

The patient was noted to have new-onset hypertension with refractory hypokalemia requiring up to 120 mmol of elemental potassium replacement daily. Raised spot urinary potassium 32 mmol/L was suggestive of urinary loss, and she had no gastrointestinal losses. Serum magnesium and thyroid function were normal. The examination did not reveal any signs and symptoms of hypokalemia or Cushing’s syndrome. Further tests confirmed adrenocorticotropic hormone (ACTH)-dependent Cushing’s syndrome (Table 1). Early morning cortisol was significantly elevated with raised ACTH level. Twenty-four-hour urinary free cortisol (UFC) was elevated at 1,464 nmol/day. Plasma renin activity and aldosterone levels were suppressed below detection levels. There was no suppression of cortisol levels with the low-dose overnight dexamethasone test. The cortisol day curve demonstrated a loss of diurnal rhythm (Figure 5). MRI brain did not report any pituitary lesion. Considering her rapid clinical presentation with severe hypokalemia and newly diagnosed lung carcinoma with other paraneoplastic syndromes, this was consistent with ectopic ACTH syndrome (EAS). The priority was to treat her hypercortisolemic state to prevent other complications. She was started on oral Ketoconazole 200 mg twice daily. Liver function tests were trended and remained normal. Her medication chart was reviewed for possible drug interactions. She was also started on *Pneumocystis jirovecii* pneumonia prophylaxis treatment. Her serum cortisol levels downtrended, and her potassium levels normalized without replacement (Figure 6).

**Table 1:** Workup for ectopic ACTH syndrome. ACTH, adrenocorticotropic hormone

Test	Result	Normal range
Serum 8 am cortisol (nmol/L)	1,268	133-537
Serum ACTH (pg/mL)	76.7	7.2-63.3
24-hour urinary free cortisol (nmol/day)	1,464	59-413
Serum 8 am cortisol post low-dose overnight dexamethasone test (nmol/L)	659	<50
Serum aldosterone (ng/dL)	<4	<21
Plasma renin activity (ng/mL per hour)	<0.6	0.6-3.0

Unfortunately, the patient deteriorated clinically and was subsequently deemed too frail for systemic chemotherapy. She was started on palliative care and demised one month after the initial diagnosis.

## Discussion

Paraneoplastic syndromes describe the remote effects of tumors that are unrelated to the physical effects of the primary or metastatic tumor. The syndromes are manifold and can present in the endocrine, neurological, dermatological, rheumatological, hematological, and nephrological systems [3]. It may precede or occur concomitantly with the diagnosis of cancer and, in some cases, herald the recurrence of the cancer.

It is known that specific syndromes are commonly associated with certain histological subtypes of lung cancer. The two most common associations are HHM in squamous cell lung carcinoma and the syndrome of inappropriate antidiuretic hormone (SIADH) secretion in SCLC [3]. As PMEC occurs so rarely, there have been no published case reports of paraneoplastic syndromes occurring in patients with PMEC. Unlike other SCLCs or NSCLCs, it remains unclear which syndromes are the most common ones associated with PMEC.

The presence of a paraneoplastic syndrome has prognostic implications. Syndromes such as Lambert Eaton Myasthenic Syndrome (LEMS) [6] and some onconeuoral antibodies such as anti-Hu antibodies portend a more favorable prognosis [7], while most other syndromes have poorer outcomes [8]. Specifically, studies have shown that HHM [9], EAS [10], and PLR [11] are all associated with shorter survival.

In particular, it has been reported that having both HHM and PLR concomitantly seems to be associated with shorter mean survival time (MST) than having hypercalcemia alone (MST 1.5 months versus 3.8 months, *P *= 0.013), but this lacked independent prognostic significance in the multivariate analysis [12]. Interestingly, another author also noted that in patients with SCLC and dual paraneoplastic syndromes of SIADH and EAS, those who presented with the syndromes sequentially have a shorter life expectancy than those who presented with both syndromes simultaneously [13].

## Conclusions

As the clinical presentation of paraneoplastic syndromes is protean, it is important for clinicians to have a high index of suspicion for and to be aware of the various syndromes, especially when the patient has a rare form of cancer. Certain syndromes such as those affecting the neurological or dermatological system may present with obvious signs and symptoms, but others such as EAS may present with more subtle signs such as refractory hypokalaemia. In many cases, identifying a paraneoplastic syndrome will also provide useful prognostic information. This case report of multiple paraneoplastic syndromes in PMEC adds to the body of literature on a rare and poorly described entity.
